# Comparative Analysis of Spray-Dried Porcine Plasma and Hydrolyzed Porcine Protein as Animal-Blood-Derived Protein Ingredients for Pet Nutrition

**DOI:** 10.3390/molecules28237917

**Published:** 2023-12-03

**Authors:** Katarzyna Kazimierska, Wioletta Biel

**Affiliations:** Department of Monogastric Animal Sciences, Division of Animal Nutrition and Food, West Pomeranian University of Technology in Szczecin, 29 Klemensa Janickiego, 71270 Szczecin, Poland; wioletta.biel@zut.edu.pl

**Keywords:** spray-dried animal plasma, proximate composition, minerals, blood products, dogs, cats

## Abstract

Spray-dried porcine plasma (SDPP) and hydrolyzed porcine protein (HPP) are promising animal protein ingredients sourced from healthy animal blood that are rich in biomolecules, including immunoglobulins, and can be an appropriate and valuable animal protein ingredient to supply the growing need for ingredients that meet the natural needs of carnivorous pets. The aim of this preliminary study was to analyze the chemical composition and mineral profile of a novel HPP compared with results for SDPP. The basic composition analysis followed AOAC guidelines, and the elemental analysis utilized atomic absorption spectrometry. Statistical comparisons employed an independent Student’s *t*-test (*p* < 0.05). Both SDPP and HPP are low in moisture (<4.3%) and rich in protein, with SDPP significantly exceeding HPP (75.4% vs. 71.4%). They boast mineral richness indicated by crude ash content (12.7% and 12.5%), featuring Na, K, P, and the trace elements Mo, Fe, and Zn. Notably, SDPP contains elevated molybdenum levels (51.39 mg/100 g vs. 10.93 mg/100 g in HPP), an essential element for diverse animal functions. Quantifying these elements in raw materials aids in achieving optimal nutrient levels in the final product. The study underscores SDPP as an excellent protein source, confirming that its nutritional value is similar to or better than other protein components in pet food.

## 1. Introduction

The latest annual report from the European Pet Food Federation [1] showed that pet ownership across Europe remains at a high level, with an estimated 91 million households in the European Union (46% of all households) owning at least one pet, of which 25% owned at least one dog. European pet owners spent more than EUR 29.1 billion on pet food, supplies, and services in 2022. Sales growth in the European pet food market was largely influenced by increased awareness of ingredients, customized food products, and grain-free and organic foods. 

**Figure 1:**
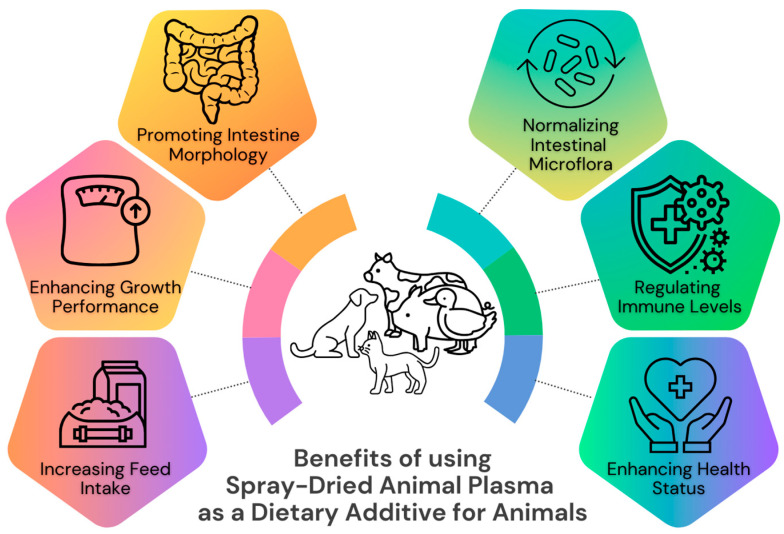
Potential health and welfare benefits of spray-dried animal plasma as a dietary additive for livestock and pets based on [2,4,5,6,7,8,9,10,11,12,13,14,15].

Pet owners are very concerned about nutrition for their pets and will purchase quality food to assure that their pets receive the proper nutrition. Dogs and cats are carnivores (facultative and obligate, respectively), which means that they need animal-origin ingredients for optimal nutrition according to their species’ requirements. Increasingly, pet foods are based on the natural diet of ancestral dogs and cats, which consisted of whole prey. Dogs and cats consume nearly all parts of their prey, including muscle, organs, bone, and blood. However, pets fed meat from grocery stores or meat added to pet food formulas are deprived of the valuable functional proteins and nutrients found in blood because it is drained from the carcass during the slaughter process. Plasma is the vital liquid component of blood that holds the blood cells in suspension and contains a diverse mixture of bioactive components with antimicrobial and immune effects that can improve animal growth and support immune function (Figure 1) [2]. Therefore, the inclusion of spray-dried animal plasma (SDAP) in commercial dog and cat food can be an appropriate and valuable animal protein ingredient to supply the growing need for ingredients that meet the natural needs of pets [3]. 

**Figure 2:**
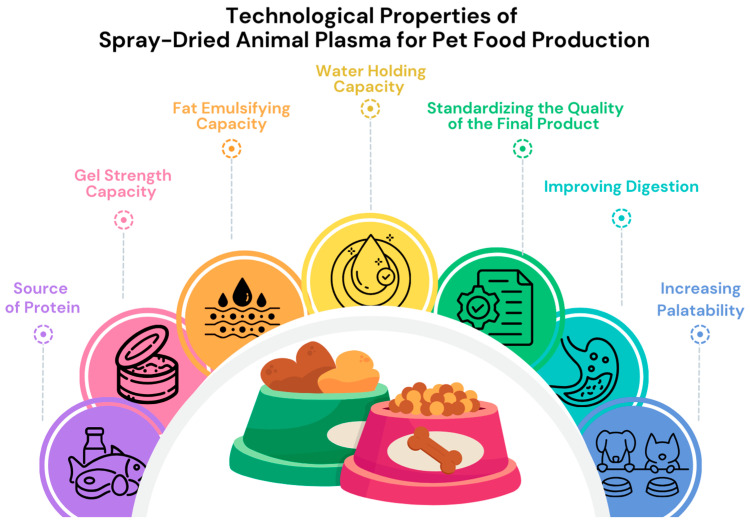
Spray-dried animal plasma in pet food manufacturing: technological and nutritional properties based on [3,17,18,19,21].

SDAP is available from different sources, including those of porcine, bovine, ovine, and avian origin, and is well recognized by the pet food industry and has applications in both wet and dry pet foods, as well as in semi-moist snacks and treats. It is used as a source of protein and has excellent gel formation and emulsifying properties, as well as a very good water-binding capacity (Figure 2) [3,16,17,18,19]. Traditionally, the pet food industry uses SDAP to improve the functional properties of wet pet food meat emulsions. According to the general opinion of pet food manufacturers, SDAP is a unique ingredient in wet formulas because it helps to standardize the quality of the final product by efficiently absorbing quality variance between batches of raw materials [18]. Due to its high (70–80%) protein content, SDAP is used to increase protein levels in raw pet food materials. Cat food containing SDAP has been shown to improve digestion [20]. In addition to their high protein content, blood products in general are also a good source of minerals and trace elements, especially iron. SDAP contains a significant amount of elements expressed as crude ash. Due to its remarkable digestibility and solubility, elements found in SDAP are highly accessible to animals. The inclusion of SDAP in a wet cat food formulation results in notable improvements in the apparent digestibility of crude ash, calcium, and phosphorus [20].

Due to its origin as a blood-derived feed ingredient, the safety of SDAP may raise concerns and therefore undergoes frequent evaluation, particularly during periods of emerging or re-emerging animal diseases across global regions. It is worth noting that it is a functional protein source obtained from the blood of healthy animals, approved by the veterinary authorities, from animals declared to be fit for slaughter for human consumption [22]. The manufacturing process of SDAP includes several steps to ensure a good microbiological quality of the product and thus its safety. Effective spray drying, utilizing rapid water evaporation that induces significant temperature and pressure changes, proves successful in inactivating crucial pathogens [23]. The low moisture content (<9%) and minimal water activity of SDAP play a pivotal role in reducing pathogen survival, particularly for bacteria and enveloped viruses, especially during extended storage [24]. To address potential viral contamination, SDAP production incorporates measures such as ultraviolet (UV) light treatment alongside the spray drying process at 80 °C throughout the substance [22]. Additionally, as an extra safety precaution, many manufacturers adopt the practice of storing SDAP at room temperature (above 20 °C) for at least 14 days before its release for sale [24,25]. Several review papers have provided detailed summaries of the production process of SDAP and its safety [22,26,27], indicating that it is an approved and safe additive in animal nutrition and pet food production.

SDAP potentially offers multiple benefits to support the health and welfare of pets, as reported for swine [4,5] and poultry and presented in Figure 1 [28,29]. Plasma contains immunoglobulin G (IgG) and other functional proteins that support the immune system [2]. In a study on rats, it was found that porcine protein concentrates, including spray-dried serum (SDS), protect against colonic inflammation in rodents and modulate immune cell function, specifically in intestinal epithelial cells (IEC18 cells) and rat spleen cells [30]. In turn, dogs fed a diet containing 12% SDAP had an increased number of total leukocytes and increased concentrations of total blood proteins and albumin [13]. In a study that measured the survival of orally administered porcine immunoglobulins in the gastrointestinal tract of adult dogs and cats fed diets containing 1% spray-dried porcine plasma (SDPP), fecal excretion of canine IgA in dogs was reduced by 41%, implying that dietary porcine γ-globulins may induce passive immunity in the canine intestine [15]. Furthermore, dog food containing SDAP has been shown to improve digestibility and reduce fecal excretion [12,13]. In addition, palatability studies showed that cats have a very high preference for a formula containing SDAP compared to a formula containing wheat gluten [18]. Therefore, SDAP is the preferred ingredient for cat food. However, SDAP used in coatings of dry dog food was neutral or lower as the first choice, although the total food intake in dogs was not different [12,13]. Spray-dried hydrolyzed porcine protein (HPP) is rich in easily digestible peptides, free amino acids, and bioactive peptides, offering functional health benefits comparable to SDAP. HPP, an enzymatically hydrolyzed plasma product, represents a high-protein source of natural origin, recommended by the manufacturer for incorporation into formulations requiring augmented palatability. Exhibiting high digestibility, HPP serves as an optimal ingredient in hypoallergenic canine diets. Utilizing an enzymatic hydrolysis process for plasma production, HPP enhances the solubility and palatability while preserving functional properties. Recently introduced for use in pet food, HPP has already been reported to be highly preferred by dogs [21]. 

Analytical control, among other things, of protein contents in animal tissues, including blood-derived products, is an important issue in fundamental and applied aspects [31]. It is important to know the nutritional composition of an ingredient before adding it to a product recipe in order to minimize the risk of deficiency or excess of particular ingredients (especially minerals) in the final product [32]. The aims of this preliminary study were to analyze the chemical composition and mineral profile of a novel hydrolyzed porcine protein (HPP) compared with results for spray-dried porcine plasma (SDPP) and discuss the nutritional value of these ingredients for applications in pet food products.

## 2. Results

The proximate composition of SDPP and HPP is shown in Table 1. SDPP and HPP are dried products with less than 4.3% water content. Both products are highly rich in protein, with SDPP being significantly higher than HPP (75.4% to 71.4%, respectively). Moreover, they are rich in minerals expressed in the form of crude ash (12.7% and 12.5%). The crude fat content is less than 1.5% for both products. The NFE was significantly higher in HPP versus SDPP. The metabolizable energy of the products was about 340 kcal in 100 g, but SDPP had significantly more calories.

SDPP and HPP significantly differed in the content of macro elements and microelements (Table 2). The greatest differences in macro elements were for potassium, where HPP contained significantly more of this element (3.88% vs. 0.55% in SDPP). For other macro elements, the largest amount was sodium (4.72% in SDPP and 2.54% in HPP), followed by phosphorus (1.88% and 1.91%), magnesium (0.01% and 0.02%), and calcium (0.01% and 0.02%). 

For the analyzed microelements, the largest amount was found for molybdenum (0.051% in SDPP and 0.011% in HPP), followed by iron (0.006% and 0.009%), zinc (0.002% for both), manganese (0.00058% and 0.000182%), and copper (0.000007% and 0.000026%).

The results were compared to the calculated protein requirements (FEDIAF [33], Table VII-11) for a 10 kg dog with moderate activity during the day. The calculations indicated that 36.90 g of SDPP or 38.98 g of HPP would be sufficient to meet the minimum protein requirement (27.84 g) of the model dog. Thus, by adding 4% SDPP to the food formula, it is possible to cover 11% of the daily protein requirements for the model dog with just this one ingredient. Based on the analyzed minerals, 3.58 g of SDPP or 6.65 g of HPP would be sufficient to meet the minimum requirement of the model dog for Na (0.17 g), 32.85 g of SDPP or 32.39 g of HPP for P (0.62 g), and 88.43 g of SDPP or 63.73 g of HPP for Fe (5 mg).

## 3. Discussion

Spray-dried animal plasma is one of many animal proteins used in wet and dry pet food. Pet food that contains SDAP is an attractive option for feeding cats and dogs because it is a natural and sustainable source of protein for carnivorous companion animals. It provides several advantages, such as good (water) binding and emulsifying properties, high palatability, and health support. Moreover, blood plasma, as a functional protein source, contains more than 697 proteins [34]. It is a complex fluid containing a variety of proteins, cytokines, growth factors, hormones, bioactive peptides, and amino acids involved in the health benefits found at the systemic and various mucosal levels when SDAP is used in animal diets [35]. 

In contrast to the extensive body of literature on the benefits of SDAP in livestock and pet nutrition, our study introduces a novel perspective by focusing on a comparative analysis of hydrolyzed porcine protein (HPP) as an alternative protein source. While SDPP has been widely recognized for its multifaceted advantages, our research examines the nutritional profile and potential benefits of novel HPP. This shift in emphasis is noteworthy, as HPP emerges as a highly preferred option in dog palatability studies [21], presenting a compelling alternative to traditional SDAP. Therefore, HPP may be used in dry kibble recipes for pets as a natural flavor enhancer to improve palatability. The use of HPP in dry pet food opens new opportunities for pet food producers to develop a diversity of new concepts, such as using HPP to improve palatability in diets oriented, for example, towards senior pets with a limited sense of smell or taste due to age.

In our analyses, SDPP had a significantly higher CP content than HPP, and it was comparable with results obtained by Zhang et al. [36], in which SDPP had the highest CP concentration (75.1%) among the five feedstuffs tested (albumen powder, 73.2%; fish meal, 67.2%; dried porcine solubles, 51.7%; spray-dried egg, 42.8%). Such a high protein content makes the 4% addition of SDPP to the dog food formula able to meet 11% of the minimum protein requirement for a model 10 kg dog. While SDPP addition to pet food has an impact on diet palatability, a 4% inclusion level of SDPP in the formula seems to be acceptable for dogs. In one preference test [13], extruded foods without or with SDPP were compared, in which the food croquettes were coated with poultry fat, then liquid and dry flavors, followed by 4% SDPP or none. The first choice of dogs was the 0% SDPP diet rather than the 4% SDPP diet (*p* < 0.05), although total intake was not different.

In general, blood products are good sources of nutrients, particularly because of their high protein and essential amino acid contents. Also, SDAP contains a significant amount of minerals expressed as crude ash (CA). Our analyses showed 12.65% CA in SDPP and 12.50% in HPP. This is in agreement with the values available in the literature for porcine blood plasma, which ranged from 7.7% [5] to 16.22% CA [37]. 

Our analysis showed less calcium in SDPP than reported by others [5,36,37], but more phosphorus and potassium. The measured amounts of phosphorus, sodium, and potassium in SDPP (1883 mg, 4718 mg, and 547 mg) and in HPP (1910 mg, 2538 mg, and 3877 mg) were higher than the values in spray-dried egg powder (81 mg, 572 mg and 519 mg, respectively) reported by Pirkwieser et al. [38]. The iron content in SDPP was 6 mg/100 g (Table 2), which is similar to the amount obtained by Sugiarto et al. [39] in whey powder but more than in other sources of protein commonly used in domestic animal feeds like fishmeal (0.24 mg/100 g [40]). However, according to Jamroz et al. [37], spray-dried porcine plasma contains approximately 10 mg of iron, i.e., similar to HPP in our analyses (8.82 mg/100 g, Table 2), which is comparable to the iron content in spray-dried whole egg powder [38]. The analyzed porcine proteins also contained more zinc (2 mg/100 g on average, Table 2) than whey protein concentrate powder (0.33 mg/100 g [41]), fishmeal (0.34 mg/100 g [40]), and the increasingly popular feed ingredient *Spirulina maxima*, where zinc was not even detected [42]. The smallest amount of microelements in the analyzed products was for copper (0.007 and 0.026 mg/100 g in SDPP and HPP, respectively), which was less than reported in spray-dried egg (0.274 mg/100 g [38]) and fishmeal (0.065 mg/100 g [40]).

Our analysis also revealed the presence of molybdenum (51.39 mg/100 g in SDPP and 10.93 mg/100 g in HPP). Molybdenum is categorized as a heavy metal and as an essential element to maintain various biochemical and physiological functions in animals. If heavy metals have atomic densities higher than 4 g/cm^3^, they also include essential elements such as zinc, copper, and iron, all of which in excess can be harmful. However, these elements are authorized as additives in animal nutrition. Molybdenum, by way of natural processes and human activities, enters the soil and water and then, via plants and livestock, can enter the food chain. Pure molybdenum is not normally added to pet food, but commonly used ingredients, including plasma, contain molybdenum. Molybdenum is an essential nutrient in animals that functions as an oxygen transfer reaction for some endogenous enzymes, but it is also known to antagonize copper absorption in ruminants [43]. There is very little information in the literature regarding molybdenum metabolism in small animals. It is crucial to maintain a sufficient supply of copper in dog food. After reviewing previous rat studies, Beynen [44] suggested that diets adequate in copper and with a molybdenum/copper ratio below 2:1 do not produce molybdenosis. Based on controlled rat studies and experimental knowledge, this suggestion can be considered both appropriate and safe for dogs and cats. There is no evidence that a deficiency or excess of molybdenum is responsible for diseases in dogs and cats. Thus, commercial pet foods appear to provide sufficient, but not excessive, molybdenum. Still, there may be an optimal range of molybdenum levels for dog and cat food, but so far there are no data on this subject. Our analysis of the molybdenum content in both ingredients (SDPP and HPP) sheds light on an underexplored aspect, offering a comprehensive understanding of the elemental composition that can influence the overall safety and efficacy of pet food. Knowing how much of each element is in the raw materials can certainly be helpful to ensure the right amounts and ratios of nutrients in the final product.

The minerals contained in SDPP are highly digestible and soluble, and therefore highly available to animals. The addition of SDAP to wet cat diets improved apparent CA, calcium, and phosphorus digestibility [20]. The higher digestibility of phosphorus as a result of the inclusion of SDAP is consistent with the high digestibility of phosphorus observed in pigs fed diets containing SDAP [45].

Moreover, SDPP contains bioactive compounds with antimicrobial and immune effects that help to improve growth efficacy and support immune function [2]. Diets containing SDPP can increase the serum IgA and IgG levels in pigs. Edwards et al. [4] found an increased serum IgG concentration (5.08 mg/mL) on day 28 and on day 68 (9.24 mg/mL) of age in piglets fed a diet with 5% SDPP. Immunoglobulins present in SDPP may balance the gut health of pets as well, binding bacteria and viruses and having an immune-modulating effect in the intestine. Andrade et al. [13] evaluated blood parameters of dogs fed diets containing increasing levels of SDPP and concluded that a diet containing 12% SDP increased the number of total leukocytes and concentrations of total blood proteins and albumin (*p* < 0.05). Regarding HPP, its novelty in the market has resulted in limited research on the subject. A recent investigation employing gilthead sea bream (*Sparus aurata*) as a model organism demonstrated that the inclusion of porcine protein hydrolysates in low fishmeal feeds led to improved growth and feed efficiency and a heightened immune response, suggesting their safety and functional efficacy [46]. Nevertheless, additional investigations across diverse species are imperative to corroborate the potential advantageous effects of HPP. Immune effects are a desired property because pets can suffer from bloating, diarrhea, and constipation.

## 4. Materials and Methods

### 4.1. Tested Products

The products used in this study were SDPP and hydrolyzed porcine protein (HPP) obtained from a commercial supplier in Spain. The powdered samples were received packaged in sealed bags. Representative samples for chemical analysis were collected and placed in sterile containers marked with successive symbols. About 200 g of each sample was used for chemical analyses conducted in triplicate.

### 4.2. Proximate Analysis

Dry matter (DM), crude protein (CP), ether extract (EE), crude fiber (CF), and crude ash (CA) were measured to assess the nutritional quality of the tested products. All tests were performed using ISO 17025 accredited methods [47] based on AOAC [48]. Samples were dried at 105 °C to a constant weight to determine dry matter (method 945.15). Crude protein (N × 6.25) (method 945.18) was analyzed by the Kjeldahl method, using a Büchi Scrubber B414 unit and a Büchi 324 distillation set (Büchi Labortechnik AG, Flawil, Switzerland). Crude fat (as an ether extract) was analyzed by the traditional Soxhlet extraction method with diethyl ether (method 2003.06). Crude fiber (method 962.09) was determined as the residue after sequential treatment with 1.25% H_2_SO_4_ and with 1.25% NaOH using an ANKOM^220^ Fiber Analyzer (ANKOM Technology, New York, NY, USA). Crude ash (method 920.153) was measured by burning in a muffle furnace at 580 °C for 8 h. Nitrogen-free extract (NFE) was calculated by the difference between the original weight of the sample and sum of the weights of its moisture, crude protein, crude fat, crude ash, and crude fiber as determined by their appropriate analysis [49].

$$NFE\left(g/100\;g\right)=100-(g\;of\;moisture+g\;of\;CP+g\;of\;EE+g\;of\;CA+g\;of\;CF)$$

### 4.3. Energy Value

On the basis of identified chemical composition, the metabolizable energy (ME, kcal/100 g) of the ingredients was calculated according to the National Research Council [50] equation using Atwater factors.

### 4.4. Macroelements Analysis

The material for macroelement (Ca, P, K, Na, Mg) concentration analyses was subjected to digestion in concentrated sulfuric acid (H_2_SO_4_) and perchloric acid (HClO_4_). Analyses for Ca, K, Na and Mg were performed using an atomic absorption spectrometer (Thermo Fisher Scientific iCE 3000 Series, Waltham, MA, USA). The following wavelengths were set to measure these minerals as follows: K, 766.5 nm; Na, 589.0 nm; Ca, 422.6 nm; Mg, 285.2 nm. The concentration of each element was calculated using a calibration curve, considering the mass of the tested portion and the dilutions used. After mineralization of the ingredient in a solution of sulfuric (VI) acid and H_2_O_2_, phosphorus (P) analysis was performed by a colorimetric method using ammonium molybdate at 470 nm. The levels of P were determined using an spectrophotometer (Specol 221, Carl Zeiss Jena, Germany). The absorbance value of the sample determined spectrophotometrically from P_2_O_5_ to the total phosphorus content was calculated according to a chemical equivalent (0.436). The reliability of the method used was confirmed by comparative studies, among other calibration curves, using the pattern series method.

### 4.5. Microelements Analysis

The material for microelement (Fe, Zn, Mn, Cu, Mo) concentration analyses was subjected to digestion in a nitric acid (HNO_3_) and perchloric acid (HClO_4_) mixture. Analyses were performed using an atomic absorption spectrometer (Thermo Fisher Scientific iCE 3000 Series, Waltham, MA, USA). The following wavelengths were set as follows to measure these minerals: Cu, 324.8 nm; Mo, 313.3 nm; Mn, 279.5 nm; Fe, 248.3 nm; Zn, 213.8 nm.

The calculation for each element concentration was performed using a calibration curve that considered the mass of the tested portion and the dilutions used. The accuracy of the analytical methods was verified based on certified reference material (CRM IAEA-A-13 Animal blood, International Atomic Energy Agency).

Results of proximate composition and macro elements were expressed as g/100 g and microelements were expressed as mg/100 g. The concentrations of CP and minerals were compared with recommendations from FEDIAF [33] considering an energy intake of 110 kcal/kg body weight (BW)^0.75^ for dogs with moderate activity (1–3 h/day).

### 4.6. Statistical Analysis

Determinations in each of the analyzed samples were performed in triplicate. Results were expressed as means ± standard deviation. Comparisons between the two types of spray-dried porcine products were made using an independent Student’s *t*-test. *p*-values < 0.05 were considered statistically significant. All statistical analyses were performed using the STATISTICA 13.0 software (TIBCO Software Inc., Palo Alto, CA, USA).

## 5. Conclusions

In conclusion, this preliminary study has confirmed that SDPP is a very good source of crude protein. The demonstrated nutritional value of SDPP and HPP is comparable to that of other protein ingredients used in pet food production. These analyzed porcine proteins are by-products of pork production, and are not specially produced food additives. They therefore contribute to maximum utilization of the pig carcass and are in line with some of the European Union’s Green Deal goals to reduce food waste and antibiotic use in livestock farming by 2030. Additionally, being products of animal origin, they are suitable for carnivores such as dogs and cats. Moreover, as indicated in previous research, these products contain biologically active components within their composition that may positively influence the immune system, among other potential benefits. Nevertheless, it is imperative for future research to delve into a comprehensive examination of bioactive compounds and their impact on animal physiology to shed more light on their potential applications.

## Figures and Tables

**Table 1:** Proximate composition (g/100 g) and energy value (kcal/100 g) of spray-dried porcine plasma and hydrolyzed porcine protein ^1^.

Item	Spray-Dried Porcine Plasma	Hydrolyzed Porcine Protein
Moisture	3.20 ^b^ ± 0.01	4.22 ^a^ ± 0.00
Crude Protein (Nx6.25)	75.43 ^a^ ± 0.28	71.42 ^b^ ± 0.62
Crude Ash	12.65 ^a^ ± 0.00	12.50 ^b^ ± 0.02
Crude Fat	1.22 ^b^ ± 0.23	1.35 ^a^ ± 0.10
NFE ^2^	7.49 ^b^ ± 0.53	10.51 ^a^ ± 0.62
ME ^3^	342.69 ^a^ ± 0.86	339.85 ^b^ ± 1.87

^1^ Values are expressed as means ± SD (*n* = 3). Different lowercase letters in the same row denote significant differences (*p* < 0.05) in means between spray-dried porcine plasma and hydrolyzed porcine protein, using a Student’s *t*-test; ^2^ NFE—nitrogen-free extract; ^3^ ME—metabolizable energy.

**Table 2:** Mineral composition of spray-dried porcine plasma and hydrolyzed porcine protein ^1^.

Item	Spray-Dried Porcine Plasma	Hydrolyzed Porcine Protein
Macroelements (g/100 g)		
Ca	0.013 ^b^ ± 0.002	0.019 ^a^ ± 0.001
P	1.883 ^a^ ± 0.000	1.910 ^a^ ± 0.037
K	0.547 ^b^ ± 0.010	3.877 ^a^ ± 0.031
Na	4.718 ^a^ ± 0.054	2.538 ^b^ ± 0.123
Mg	0.014 ^b^ ± 0.001	0.022 ^a^ ± 0.005
Microelements (mg/100 g)		
Cu	0.007 ^b^ ± 0.001	0.026 ^a^ ± 0.001
Fe	6.359 ^b^ ± 0.413	8.824 ^a^ ± 0.003
Mn	0.508 ^a^ ± 0.039	0.182 ^b^ ± 0.019
Zn	1.910 ^b^ ± 0.005	2.040 ^a^ ± 0.036
Mo	51.389 ± 0.302	10.925 ^b^ ± 0.186

^1^ Values are expressed as means ± SD (*n* = 3). Different lowercase letters in the same row denote significant differences (*p* < 0.05) in means between spray-dried porcine plasma and hydrolyzed porcine protein, using a Student’s *t*-test.

## Data Availability

All data generated or analyzed during this study are included in this published article.
